# The Effects of Acceptance and Commitment Therapy (ACT) Intervention on Inflammation and Stress Biomarkers: a Randomized Controlled Trial

**DOI:** 10.1007/s12529-020-09891-8

**Published:** 2020-05-11

**Authors:** Elina Järvelä-Reijonen, Sampsa Puttonen, Leila Karhunen, Essi Sairanen, Jaana Laitinen, Mikko Kolehmainen, Jussi Pihlajamäki, Urho M Kujala, Riitta Korpela, Miikka Ermes, Raimo Lappalainen, Marjukka Kolehmainen

**Affiliations:** 1 Institute of Public Health and Clinical Nutrition, Clinical Nutrition, University of Eastern Finland, P.O. Box 1627, FI-70211 Kuopio, Finland; 2 Finnish Institute of Occupational Health, P.O. Box 40, FI-00251 Helsinki, Finland; 3 Institute of Clinical Medicine and Clinical Nutrition, Kuopio University Hospital, P.O. Box 100, FI-70029 KYS Kuopio, Finland; 4 Department of Psychology, University of Jyväskylä, P.O. Box 35, FI-40014 Jyväskylä, Finland; 5 Department of Social and Psychological Studies, Karlstad University, SE-651 88 Karlstad, Sweden; 6 Department of Environmental and Biological Sciences, University of Eastern Finland, P.O. Box 1627, FI-70211 Kuopio, Finland; 7 Faculty of Sport and Health Sciences, University of Jyväskylä, P.O. Box 35, FI-40014 Jyväskylä, Finland; 8 Medical Faculty, Pharmacology, Medical Nutrition Physiology and Human Microbe Research Program, University of Helsinki, P.O. Box 63, FI-00014 Helsinki, Finland; 9 VTT Technical Research Centre of Finland, P.O. Box 1300, FI-33101 Tampere, Finland

**Keywords:** Psychological flexibility, ACT, Mindfulness, Obesity, Low-grade inflammation, Stress

## Abstract

**Background:**

Psychological processes can be manifested in physiological health. We investigated whether acceptance and commitment therapy (ACT), targeted on psychological flexibility (PF), influences inflammation and stress biomarkers among working-age adults with psychological distress and overweight/obesity.

**Method:**

Participants were randomized into three parallel groups: (1) ACT-based face-to-face (*n* = 65; six group sessions led by a psychologist), (2) ACT-based mobile (*n* = 73; one group session and mobile app), and (3) control (*n* = 66; only the measurements). Systemic inflammation and stress markers were analyzed at baseline, at 10 weeks after the baseline (post-intervention), and at 36 weeks after the baseline (follow-up). General PF and weight-related PF were measured with questionnaires (Acceptance and Action Questionnaire, Acceptance and Action Questionnaire for Weight-Related Difficulties).

**Results:**

A group × time interaction (*p* = .012) was detected in the high-sensitivity C-reactive protein (hsCRP) level but not in other inflammation and stress biomarkers. hsCRP decreased significantly in the face-to-face group from week 0 to week 36, and at week 36, hsCRP was lower among the participants in the face-to-face group than in the mobile group (*p* = .035, post hoc test). Age and sex were stronger predictors of biomarker levels at follow-up than the post-intervention PF.

**Conclusion:**

The results suggest that ACT delivered in group sessions may exert beneficial effects on low-grade systemic inflammation. More research is needed on how to best apply psychological interventions for the health of both mind and body among people with overweight/obesity and psychological distress.

**Trial Registration:**

ClinicalTrials.gov Identifier: NCT01738256, Registered 17 August, 2012

## Introduction

Chronic low-grade systemic inflammation is a minor activation of the inflammatory system, without actual infection or tissue injury [1], which is present in several metabolic dysfunctional states such as obesity, type 2 diabetes, and cardiovascular disease [2, 3]. In low-grade systemic inflammation, the circulating levels of C-reactive protein (CRP) and interleukin-1 receptor antagonist (IL-1Ra) are increased whereas the levels of anti-inflammatory markers, such as adiponectin, are decreased [4]. Elevated high-sensitivity C-reactive protein (hsCRP) and IL-1Ra levels and low adiponectin levels predict a persistence of the metabolic syndrome [5]. A chronic low-grade systemic inflammation also often co-occurs with a chronic stress response [6].

Stress is a complex concept with no uniform definition [7]. The response to a stressful situation is usually both psychological and physiological [8, 9]. The physiological stress response, i.e., the activation of the hypothalamic-pituitary-adrenocortical (HPA) axis and sympathetic nervous system [10], leads to altered secretion of cortisol and dehydroepiandrosterone sulfate (DHEAS) [11, 12]. Cortisol and DHEAS are markers of HPA axis activation [12] and, thus in this study, are exploited as markers of either acute or chronic stress reactions [11–14]. Altered cortisol secretion is associated with, for example, immune and inflammatory outcomes, obesity, cancer, and increased risk for mortality [15]. DHEAS is known to counteract several of the effects of cortisol and to, for example, attenuate inflammatory process and reduce mortality [12, 16, 17]. Allostatic load is a holistic concept of how chronic stress can lead to negative health outcomes [18, 19]. Markers of inflammation and HPA axis activity are suggested to mediate the effects of chronic stress underpinning disturbed health [14, 20, 21]. Thus, finding ways to improve the circulating levels of the inflammatory and stress biomarkers would be one approach to improving long-term health.

In addition to chronic stress, also obesity and lifestyle factors such as diet and physical activity are related to low-grade systemic inflammation [22]. Adipose tissue is a major source of stress-related pro-inflammatory markers [22]. Conversely, a diet consistent with nutrition recommendations, such as a healthy Nordic diet, is associated with better inflammatory status [23, 24].

Obesity, psychosocial distress, a diet not following nutritional recommendations, and a sedentary lifestyle are major health challenges in our modern society. For an individual, these challenges are difficult to overcome—it is not easy to make long-term lifestyle changes. For example, the majority of individuals tend to regain weight after weight loss [25]. Often, these difficulties arise from our problematic inner experiences and psychological processes. For example, individuals who gain weight after they have lost weight tend to escape or avoid their problems [26] and adopt an inflexible, dichotomous thinking style [27, 28]. Psychological inflexibility means that the person is not able to change or maintain a behavior which would lead to personally valued outcomes and is not able to fully contact the present moment [29]. The non-adaptive psychological processes, such as psychological inflexibility [29], are thought to underlie a wide variety of forms of human suffering [30], irrespective of diagnoses [31]. Psychological inflexibility is also associated with higher uncontrolled and emotional eating [32] which are features of eating behavior associated with poorer dietary choices and higher BMI [33–35]. Thus, increasing psychological flexibility, i.e., the ability to contact the present moment and to change or persist in behavior according to personal values [29], could be one possible way to promote an individual’s health and well-being.

One of the so-called process-based therapies aimed at improving psychological flexibility in general is acceptance and commitment therapy (ACT) [36]. ACT seeks to increase psychological flexibility and consists of six interrelated core processes: (1) clarification of own values, (2) commitment to act based on those values, (3) being in contact with the present moment (i.e., mindfulness), (4) having self as context (i.e., being aware of thoughts, feelings, etc., without attaching to them), (5) defusion (i.e., altering the way to interact with or relate to thoughts, feelings, etc.), and (6) acceptance [29].

Previous studies have investigated the effects of ACT or value clarification interventions on the cortisol response in individuals performing an experimental psychosocial stress test [37, 38]. To the best of our knowledge, there are no previous studies about the effectiveness of an ACT intervention on inflammation and stress biomarkers in real-world non-clinical, randomized controlled settings. However, interventions based on one part of ACT, namely mindfulness, have shown positive effects on some inflammation and stress biomarkers such as hsCRP [39, 40] and cortisol [41]. However, the reviews of mindfulness-based randomized controlled interventions have concluded that the effects on circulating inflammation markers [42–44] and cortisol [44] are contradictory, and warrant further research.

In summary, based on the literature, it can be hypothesized that problems in psychological processes (i.e., psychological inflexibility) are closely related to behavior (e.g., poor diet and sedentary lifestyle leading to obesity) and subjective psychosocial distress. All these factors are linked to chronic low-grade systemic inflammation and a chronic physiological stress response. These physiological processes, in turn, are a risk of future ill health and major chronic diseases. The aim of this study was to investigate how the effects of an ACT intervention, targeting psychological inflexibility, can alter inflammation and stress biomarkers among adults with psychological distress and overweight. In addition, because the psychological and physiological factors are intertwined, we conducted post hoc, exploratory analyses to explore how psychological flexibility (general or weight-related) and the biomarkers are clustered within this study population or whether they cluster more strongly with other psychological, anthropometric, or lifestyle (diet, physical activity) measures.

## Methods

### Study Design

The present study is a secondary analysis of a parallel-arm randomized controlled trial, in which three different psychological interventions were studied and compared to a non-treatment control group [45]. The randomization process and CONSORT flow chart have been published previously [45]. The present study investigates the effects of the two ACT-based intervention arms compared to control.

The study participants were recruited by advertisements in local newspapers and screened for eligibility via telephone inquiry and an online questionnaire from August 2012 until January 2013. The participants had to be 25–60 years old and have a self-reported body mass index (BMI) of 27–34.9 kg/m^2^. This range for self-reported BMI was used because we aimed to have participants with overweight and obesity and anticipated that the range of laboratory-measured BMI would be most likely wider. The participants also had to be psychologically distressed (≥ 3/12 points from the General Health Questionnaire (GHQ-12) [46]) and have computer and Internet access. There were several exclusion criteria, such as diagnosed severe chronic illness (including symptomatic cardiovascular disease, type 1 or 2 diabetes, kidney disease requiring dialysis, and eating disorder), disabilities/illnesses substantially affecting physiological or mental health, medical surgery within the past 6 months, heart attack or stroke within the past 6 months, pacemaker, regular oral cortisone medication, pregnancy or breastfeeding within the past 6 months, psychotherapy or other psychological or mental treatments at least twice a month, disability pension for psychological reasons, and participation in some other intervention trial during the present study. An additional exclusion criterion for the present analyses was a hsCRP value > 10 mg/L indicating acute infection or inflammation [47]. The study was conducted in three cities in Finland (Jyväskylä, Kuopio, and Helsinki), and the recruitment was conducted in two phases. The participants in the first phase started in autumn, and the second phase started in spring. The participants filled in electronic questionnaires, visited the local study center for clinical and biochemical measurements, and reported their food consumption in a 48-h dietary recall by telephone. Measurements were conducted before the intervention (baseline, study week 0), after the 8-week intensive intervention period (post-intervention, study week 10), and 36 weeks after baseline measurements (follow-up, study week 36). The measurements were collected from August 2012 until December 2013.

The sample size of the current study is based on the power calculation (for depression symptoms) of the randomized controlled trial [45], resulting in a sample size of *n* = 80–85 per group.

### Study Groups

The face-to-face and mobile interventions were based on the same ACT program constructed by the same research group. Thus, only the delivery method of the intervention differed. The two interventions included the following main components: value clarification, acting according to one’s own values, mindfulness skills, the observing self (e.g., observing thoughts without being caught up in them), and acceptance skills (e.g., making room for unpleasant feelings and urges, allowing them to come and go). The main focus was on ACT skills, but minor parts of mindful eating, relaxation, and everyday physical activity were also included. However, the intervention did not include nutrition education. Only a hyperlink to a public nutritional website was provided to the participants in the intervention groups, which was to be utilized if the dietary changes were according to one’s own values. Lappalainen et al. [45] for a more detailed description of the intervention.

The face-to-face group had six group sessions led by a psychologist during the 8-week intervention period. Each session took approximately 90 min, and each group consisted of 6–12 participants. The sessions included exercises, pair and group discussions, and homework for which the participants received a workbook. The treatment adherence has been described previously and was found to be good [48]. On average, the participants attended five group sessions (SD = 1).

The mobile group had one group session in which participants learned about the principles of ACT and received smartphones with the pre-installed Oiva mobile app [49]. The Oiva app contains 46 exercises in text and audio formats and introductory videos about the ACT skills. The user experience results of the app were positive [49]. The participants were free to choose exercises and videos in any order and to do them as many times as the participants wanted during the 8-week intervention period. The participants returned the smartphones during the post-intervention laboratory study visit. The participants’ usage of the mobile app has been reported in detail previously [50]. Briefly, among the study population of the present analyses, the median total duration of the mobile app use was 280 min (interquartile range 198–423, total range 52–2001).

Participants randomized to the control group took part in all of the measurements but did not receive any intervention. After the follow-up measurements, the participants in the control group had an opportunity to attend one group session in which the principles of ACT were presented and they were told that they could utilize an Internet-based lifestyle coaching program [45].

### Measures

#### Inflammation and Stress Biomarkers

An antecubital venous blood sample was taken after a 12-h overnight fast in the study laboratory between 7 and 10 a.m. The plasma samples were collected into EDTA tubes and centrifuged as soon as possible. The serum samples were centrifuged after the blood had clotted. The samples were stored at − 80 °C until analyzed.

Inflammation markers known to be associated with metabolic syndrome (MetS) components (i.e., high hsCRP and IL-1Ra levels and low adiponectin level) [51] were analyzed. Plasma hsCRP concentration was determined with a photometric immunoturbidimetric method (Konelab; Thermo Fisher Scientific, Vantaa, Finland), with a measurement range from 0.1 to 10 mg/L, and extended range using automatic dilution from 0.1 to 40 mg/L. The level of plasma IL-1Ra was measured with an enzyme immunoassay (Quantikine^®^ ELISA Kits; R&D Systems, Inc., Minneapolis, USA) with a measurement range from 31.2 to 2000 pg/mL. Serum high molecular weight (HMW) adiponectin was measured with enzyme immunoassay (Quantikine^®^ ELISA for Human HMW Adiponectin/Acrp Immunoassay; R&D Systems, Inc., Minneapolis, USA) with a measurement range from 0.39 to 25 μg/mL.

Plasma total cortisol concentrations were measured with chemiluminescent immunoassay (LIAISON^®^ Cortisol; DiaSorin, Saluggia, Italy) with a quantitation limit of 4.1 nmol/L and a dilution threshold of 2208 nmol/L. Plasma DHEAS was determined with chemiluminescent immunoassay (LIAISON^®^ DHEA-S; DiaSorin, Saluggia, Italy) with a quantitation limit of 0.027 μmol/L and a dilution threshold of 20.3 μmol/L. The cortisol/DHEAS ratio was used as a more sensitive index of the HPA axis activation [12] and the catabolic/anabolic balance [14, 52] under stress conditions. The cortisol/DHEAS ratio was calculated by dividing the raw value of cortisol (ng/mL) by the raw value of DHEAS (ng/mL) [52]. The ratio between cortisol and DHEAS, i.e., the balance of these catabolic and anabolic stress hormones, is suggested to be more informative of psychiatric and general health status than either of the hormone levels alone [14]. Although the DHEAS concentration increases as a part of an acute stress response [14], its levels decline during chronic stress conditions [12, 14]. DHEAS has been related to several positive health effects [17] and has been shown to counteract several of cortisol’s effects [12, 16]. Thus, a higher cortisol/DHEAS ratio is proposed to indicate higher chronic stress and to contribute to ill health [14].

#### Anthropometric Measurements

Weight and height were measured with calibrated instruments in the study laboratory in the morning after a 12-h overnight fast. BMI was calculated as kilograms per meters squared. Waist circumference was measured halfway between the lowest rib and the iliac crest. Body composition (% of body fat) is based on multifrequency bioelectrical impedance analysis using an In-Body 720 device (Mega Electronics, Kuopio, Finland) or Tanita BC-418 MA device (Tanita, Japan).

#### Psychological Flexibility

Psychological flexibility was measured with two questionnaires. The Acceptance and Action Questionnaire (AAQ-II) [53] measures general psychological flexibility. The 7 items (e.g., “I worry about not being able to control my worries and feelings”) are answered with 7-point Likert scale from “never true” (1) to “always true” (7). The possible score range is 7–49 with a lower score reflecting more psychological flexibility. Cronbach’s alpha was 0.91.

The Acceptance and Action Questionnaire for Weight-Related Difficulties (AAQW) [54] measures weight-related psychological flexibility. The 22 items (e.g., “I try hard to avoid feeling bad about my weight or how I look”) are answered with a 7-point Likert scale from “never true/not at all believable” (1) to “always true/completely believable” (7). The possible score range is 22–154. Here too, a lower score reflects more psychological flexibility related to difficult weight-related thoughts and feelings. Cronbach’s alpha was 0.90.

#### Psychological Stress and Symptoms of Depression

The 12-item General Health Questionnaire (GHQ-12) [46] was used to screen the volunteers for psychological distress. The GHQ-12 has been found to be a valid screening tool for common mental health problems in the Finnish population [55]. The respondents were asked, considering the past few weeks, to answer questions such as “Have you recently felt constantly under strain?” In the screening, a bimodal scoring system was used (“not at all” (0 point), “same as usual” (0), “rather more than usual” (1), and “much more than usual” (1), with the total sum score ranging from 0 to 12). Cronbach’s alpha was 0.73. In the present analyses, Likert scoring (0, 1, 2, and 3 points; possible range 0–36) was used in the statistical analyses to achieve a larger variation in the GHQ-12 scores. Cronbach’s alpha using Likert scoring was 0.82. The 14-item Perceived Stress Scale (PSS-14) [56] was used to assess the degree to which a person perceives life as being stressful. The questionnaire has demonstrated acceptable psychometric properties worldwide [57]. Questions concern how often a person has experienced certain feelings and thoughts during the previous month, e.g., “In the last month, how often have you found that you could not cope with all the things that you had to do?” The 5-point Likert scale ranged from “never” (0) to “very often” (4) and is summed for the total score (possible range 0–56). Cronbach’s alpha was 0.88. Symptoms of depression were measured by the 21-item Beck Depression Inventory-II (BDI-II) [58]. The 4-point Likert scale is scored from 0 to 3, and the scores are summed to calculate the total score (possible range 0–63). Cronbach’s alpha was 0.87. For all these measures, higher scores indicate higher psychological distress and depressive symptoms.

#### Diet and Physical Activity

Index of Diet Quality (IDQ) questionnaire [59] measured adherence to Nordic and Finnish nutrition recommendations. Apparently unrealistic answers (e.g., 27 slices of bread per day) were verified from the participant when possible or coded as missing. Alcohol consumption during the previous 6 months was assessed using Alcohol Use Disorders Identification Test Consumption (AUDIT-C) questionnaire [60]. A 48-h dietary recall was conducted on the telephone between Tuesday and Friday. The time for the telephone call was pre-scheduled for practical reasons, and the participants were informed that the interviewer would ask about their diet. However, it was not mentioned that the interview will consider their diet during the past 48 h. The interview protocol was planned based on the protocol used in national FINDIET 2012 Survey [61]. We placed a special emphasis on the protocol to assist the participant to remember and give accurate information of the foods and drinks consumed. The protocol has been described in more detail previously [62]. Nutrient intake was calculated based on the 48-h dietary recall using AivoDiet software (v 2.0.2.2; Aivo Ltd., Turku, Finland), utilizing the Fineli^®^ Finnish Food Composition Database (National Institute for Health and Welfare, Nutrition Unit, Helsinki, Finland).

Leisure time physical activity and commuting activity were assessed by a questionnaire [63, 64]. Leisure time metabolic equivalent (MET) index (MET h/day) was calculated as a sum score of the different activities multiplied by the intensity (MET), duration (h), and frequency of the activity [63, 64].

### Statistical Methods

Statistical analyses were conducted with IBM SPSS Statistics (version 23) and MATLAB R2017b. A *p* value < .05 was considered as statistically significant.

Baseline differences between the groups were analyzed using Pearson chi-square test for categorical variables and one-way ANOVA with the Tukey HSD post hoc test for continuous variables. The normality assumption was assessed by the histograms of the residuals. If the normality assumption was not met, the non-parametric Kruskal-Wallis test was used. In the results, descriptive values of the normally distributed variables are presented as means ± SD and of the non-normally distributed variables as medians (interquartile range).

General linear mixed model was used to analyze the differences between the three study groups using all three time points (group × time interaction) and the main effect of time on outcome variables. Participants were included as random effects and intercept, group, time, interaction term, and covariates as fixed effects. In case of a statistically significant group × time interaction, the Sidak post hoc test was conducted for pairwise comparisons. The analysis utilizes all of the available data with the assumption of missing data as “missing at random.” The missing data also seemed to be “missing at random” as there were no differences in the study group, starting time of the study, study center (chi-square test, *p* = .303, *p* = .460, and *p* = .496, respectively), gender (Fisher’s exact test, *p* = .302), age, GHQ-12 score, and baseline BMI (*t* test, *p* = .548, *p* = .374, and *p* = .141, respectively) between the participants who provided all data as compared to the participants with missing data. Participants considered as outliers (over mean ± 5 SDs) at any of the three time points were excluded. The distributions of the values were so wide that using the cutoff of mean ± 5 SDs enabled to exclude the true, explicit outliers. The normality assumption was assessed by the residual histograms. The non-normally distributed outcome variables hsCRP, IL-1Ra, HMW adiponectin, and cortisol/DHEAS ratio were log-transformed. All the analyses were adjusted for study center and starting time of the study (i.e., basic adjusted model) and, in a fully adjusted model, also for age, sex, and baseline BMI. Furthermore, to study the effect of baseline BMI on the intervention effects, a group × time × baseline BMI interaction term was added into the model.

Linear regression analysis was used to investigate whether psychological flexibility (general or weight-related) after the intervention (week 10) predicted the levels of inflammation and stress biomarkers after the follow-up period (week 36) among the participants in the ACT intervention groups. At first, psychological flexibility, age, sex, study center, and starting time of the study were included in the model as independent variables. Because study center and starting time of the study were non-significant in each model, they were excluded from the final models. The assumptions were evaluated with the Durbin-Watson test (between 1 and 3), tolerance (0.1–1), VIF (1–10), studentized residuals (about 95% between − 2 and 2), Cook’s distance < 1, linearity in scatter plots, and normality in residual histograms [65]. All the models met the assumptions.

Exploratory principal component analyses (PCAs) were conducted for psychological, physiological, anthropometric, and lifestyle measures. These factors are often interrelated, but we wanted to explore if the measures were clustered in this sample. Two analyses were performed: (a) with values at baseline and (b) for changes from baseline to week 36 (Δ36 = week 36 value − week 0 value). The PCAs were intended to be descriptive and hypothesis generating instead of being formal hypothesis testing. The intention of these post hoc PCAs was to explore how psychological flexibility (general or weight-related) and the biomarkers would cluster among the study participants: do they correlate with each other or correlate more strongly with other psychological, anthropometric, and lifestyle measures. In the PCA with the changes from baseline to week 36, missing data was handled by excluding the cases with any missing data. Of the 200 participants with data from both time points, 11% (*n* = 21) had to be excluded because of hsCRP > 10 and 3% (*n* = 5) were excluded because of a missing value in any of the variables resulting in 174 participants providing full data for this analysis. An outlier with a value over mean ± 5 SDs was excluded (*n* = 1 because of a high increase in HMW adiponectin value) resulting in *n* = 173. PCAs were conducted with standardized values. The Humphreys-Ilgen parallel analysis was used to determine the number of components [66, 67]. The components were rotated using varimax rotation [68]. Loadings of the rotated components are reported. A threshold of 0.3 was used for interpreting the baseline components’ loadings. A threshold of 0.25 was used for interpreting the change (Δ36) components’ loadings in order to include the inflammation markers into the components.

## Results

### Participants

Of the 254 individuals randomized to the face-to-face (*n* = 84), mobile (*n* = 85), and control (*n* = 85) groups, 219 participated in baseline measurements (Table 1). At baseline, 13 participants (6%) had a hsCRP value > 10 mg/L indicating acute infection or inflammation [47] and were excluded from the present analyses. For the same reason, nine participants (4%) were excluded from the week 10 and 15 participants (8%) from week 36 assessments. In addition, one participant was excluded from the analyses at each measurement time point (different persons each time) because of a high IL-1Ra value considered to be an outlier (over 5 SDs above the mean) and one participant (same person each time) because of a very high adiponectin value (over 5 SDs above the mean). The numbers of participants included in the present analyses are shown in Tables 1 and 2.

**Table 1:** Characteristics of the participants in each group at baseline (*n* = 204)

	Face-to-face	Mobile	Control	*p*^a^
Number of participants (*n*)				
Randomized	84	85	85	
Participated in baseline measurements	70	78	71	
Included in the present analyses	65	73	66	
Starting time of the study (*n*)				
Autumn^b^	32	34	29	.832
Spring^c^	33	39	37	
Study center (*n*)				
Jyväskylä	18	20	16	.985
Kuopio	21	25	22	
Helsinki	26	28	28	
Sex (*n*)				
Female	56	61	54	.795
Male	9	12	12	
Age (years)	51.0 ± 6.5	48.8 ± 7.7	49.0 ± 7.5	.148
Weight (kg)	85.9 ± 10.4	88.3 ± 10.4	88.5 ± 11.5	.308
BMI (kg/m^2^)	30.8 ± 3.0	31.5 ± 2.7	31.3 ± 2.9	.405
Waist circumference (cm)	101.6 ± 9.0	103.3 ± 7.9	103.5 ± 9.1	.407
Psychological distress (GHQ-12 score)	7.0 (4.5–9.5)	6.0 (5.0–9.0)	7.0 (5.0–10.0)	.564^d^
Perceived stress (PSS score)	25.8 ± 8.0	26.9 ± 7.7	27.1 ± 7.6	.558
General psychological flexibility (AAQ-II score)	19.9 ± 8.6	20.2 ± 9.2	21.5 ± 9.3	.527
Weight-related psychological flexibility (AAQW score)	84.3 ± 19.3	87.7 ± 21.1	87.8 ± 21.2	.540
Diet quality (IDQ score)	10.4 ± 2.1	10.5 ± 1.9	10.2 ± 2.1	.620
Leisure time physical activity (MET index)	3.6 ± 2.9	3.3 ± 3.2	2.9 ± 3.0	.435

Values are *n* / mean ± SD / median (interquartile range)

*BMI* body mass index, *GHQ-12* General Health Questionnaire-12, *PSS* Perceived Stress Scale, *AAQ-II* Acceptance and Action Questionnaire, *AAQW* Acceptance and Action Questionnaire for Weight-Related Difficulties, *IDQ* Index of Diet Quality, *MET* metabolic equivalent

^a^*p* value for differences between the study groups (Pearson chi-square for categorical variables and one-way ANOVA for continuous variables unless otherwise noted)

^b^September–October 2012

^c^January–February 2013

^d^Non-parametric Kruskal-Wallis test

**Table 2:** The effects of the ACT-based face-to-face and mobile interventions on inflammation and stress biomarkers (*n* = 192) analyzed with the linear mixed model

	Face-to-face			Mobile			Control			*p*^a^ (group × time)	*p*^a^ (time)
	0 week (*n* = 64)	10 weeks (*n* = 57)	36 weeks (*n* = 55)	0 week (*n* = 67)	10 weeks (*n* = 64)	36 weeks (*n* = 62)	0 week (*n* = 61)	10 weeks (*n* = 58)	36 weeks (*n* = 57)		
hsCRP (mg/L)^b^	1.2 (0.6–2.3)	1.5 (0.5–2.6)	1.2 (0.4–2.0)	1.6 (0.5–3.7)	1.6 (0.9–2.7)	1.7 (0.8–3.3)	1.5 (0.5–3.0)	1.4 (0.6–2.9)	1.5 (0.6–3.3)	*.012*	.391
										*.012*	.406
IL-1Ra (pg/mL)^b^	356.5 (258.8–528.1)	383.6 (263.3–522.3)	301.1 (248.5–425.5)	366.6 (265.9–607.8)	366.1 (258.3–559.4)	333.1 (246.5–482.1)	417.1 (277.2–572.8)	387.0 (281.7–594.8)	367.1 (292.5–526.3)	.883	*< .001*
										.880	*< .001*
HMW adiponectin (μg/mL)^b^	4.9 (2.8–7.3)	4.9 (3.2–6.7)	5.1 (2.8–7.8)	5.0 (3.3–7.8)	4.7 (3.3–7.1)	4.8 (3.0–7.4)	4.8 (3.1–6.7)	4.7 (3.0–6.9)	4.8 (3.0–7.1)	.353	.848
										.353	.877
Cortisol (ng/mL)	115.6 ± 45.9	109.7 ± 39.2	117.3 ± 42.6	123.1 ± 47.9	117.3 ± 42.5	118.5 ± 39.0	108.9 ± 35.8	116.1 ± 41.4	119.2 ± 40.3	.272	.467
										.287	.466
DHEAS (ng/mL)	982.3 ± 518.4	969.1 ± 468.5	952.7 ± 495.0	1013.7 ± 540.0	1015.1 ± 547.2	997.3 ± 493.4	939.4 ± 424.6	951.7 ± 440.6	908.3 ± 440.6	.887	.093
										.884	.098
Cortisol/DHEAS ratio^b^	0.12 (0.09–0.19)	0.12 (0.08–0.17)	0.13 (0.07–0.20)	0.12 (0.08–0.19)	0.12 (0.08–0.18)	0.13 (0.08–0.20)	0.12 (0.09–0.16)	0.13 (0.09–0.16)	0.13 (0.10–0.21)	.129	.053
										.119	.056

The values are unestimated medians (interquartile range) / means ± SD (i.e., without adjustments). There were no statistically significant differences (*p* values > .186) in the baseline values between the groups

*hsCRP* high-sensitivity C-reactive protein, *IL-1Ra* interleukin-1 receptor antagonist, *HMW* high molecular weight, *DHEAS* dehydroepiandrosterone sulfate

^a^*p* value for differences between the three study groups using all measured time points (study weeks 0, 10, and 36) analyzed with the linear mixed model. The *p* value above is adjusted for study center and starting time of the study, and the *p* value below is adjusted for study center, starting time of the study, age, sex, and baseline BMI. Values in italics indicate a significant *p* value < 0.05

^b^Analyzed with logarithmically transformed values. Values presented in the table are non-transformed

The measured BMI of the participants at the baseline laboratory visit (*n* = 204, 84% females) ranged between 25.7 and 40.1 kg/m^2^ (mean ± SD 31.2 ± 2.9 kg/m^2^). Age ranged between 27 and 61 years (50 ± 7 years). Less than a third (28%) had a high relative risk for cardiovascular disease (hsCRP > 3.0 mg/L), whereas 36% had hsCRP levels reflecting low risk (< 1 mg/L) and 36% reflecting average (1.0 to 3.0 mg/L) risk [69]. There were no differences in the baseline demographic, clinical, psychological, and lifestyle characteristics between the three study groups (Table 1).

### Intervention Effects on Inflammation and Stress Biomarkers

**Fig. 1:**
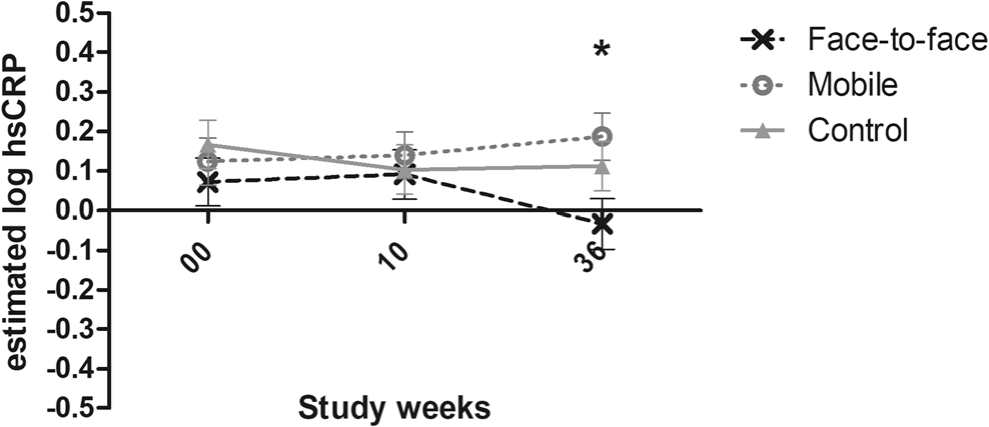
The effects of ACT intervention on logarithmized hsCRP adjusted for study center and starting time of the study. The values are log-transformed estimated (adjusted for study center and starting time of the study) marginal means ± standard error (SE). The measurements were conducted before the intervention (baseline, study week 0), after the 8-week intervention period (study week 10), and 36 weeks after the baseline measurements (study week 36). The group × time interaction for the entire study period was significant (*p* = .012). The face-to-face and mobile groups had a significant difference at week 36 (*p* = .035, post hoc test, indicated with an asterisk). The decrease of hsCRP from week 0 to week 36 and from week 10 to week 36 was significant in the face-to-face group (*p* = .045 and *p* = .014, respectively)

During the entire study period, a group × time interaction was found in hsCRP but not in the other biomarkers (Table 2). In the basic adjusted model, the level of hsCRP decreased significantly in the face-to-face group from week 0 to week 36 (*p* = .045 for the post hoc test) and from week 10 to week 36 (*p* = .014, post hoc test). The only difference between the groups was that the hsCRP was lower among the participants in the face-to-face group as compared to the mobile group in the week 36 (*p* = .035, post hoc test) with a small-to-medium effect size (*r* = − .196, *p* = .035) (Fig. 1). Thus, the ACT groups did not differ from the control group significantly. In the fully adjusted model, the difference between the face-to-face and mobile groups was no longer apparent in week 36, with merely a trend (*p* = .062, post hoc test) being detected. In this model, baseline BMI was significant (estimate .054, *p* < .001). However, the decrease in hsCRP from week 0 to week 36 (*p* = .048, post hoc test) and from week 10 to week 36 (*p* = .014, post hoc test) remained significant in the face-to-face group. The hsCRP values presented in Table 2 are the actual unestimated values (i.e., without adjustments) for clinical interpretation. Figure 1 shows the logarithmically transformed estimated (adjusted for study center and starting time of the study) values from the statistical analysis. The estimated marginal mean ± SE hsCRP values in the face-to-face group for weeks 0, 10, and 36 without logarithmic transformation were 1.955 ± 0.253 mg/L, 2.150 ± 0.261 mg/L, and 1.759 ± 0.264 mg/L, respectively.

During the entire study period, a significant decrease (*p* < .001) in IL-1Ra over time was evident, in all of the study groups (Table 2). The logarithmically transformed estimated marginal mean ± SE IL-1Ra values for weeks 0, 10, and 36 of the fully adjusted model were 2.588 ± .014, 2.597 ± .014, and 2.555 ± .014, respectively. In addition, a trend (*p* = .056) towards an increase in the cortisol/DHEAS ratio was observed (log-estimated marginal mean ± SE values for weeks 0, 10, and 36 of the fully adjusted model − .887 ± .019, − .893 ± .019, and − .864 ± .019, respectively).

There were no statistically significant differences (*p* values > .186) in the baseline values of the inflammation and stress biomarkers between the groups (Table 2). The intervention effect was not dependent on baseline BMI (group × time × baseline BMI interaction non-significant for all inflammation and stress biomarkers).

### Association of Psychological Flexibility with Inflammation and Stress Biomarkers

We further analyzed whether the main target of the ACT intervention, psychological flexibility, was associated with the levels of inflammation and stress biomarkers among the participants in the ACT intervention groups (face-to-face and mobile) over the long term. During the intensive intervention period (from baseline to week 10), general psychological flexibility increased in most of the participants (54%), but it decreased in 31%, while remaining the same in 15% of the participants in the ACT groups. Weight-related psychological flexibility increased in 66%, decreased in 33%, and remained the same in 1% of the participants in the ACT groups. The effects of the intervention on psychological flexibility have been reported previously [70].

The level of psychological flexibility (general or weight-related) after the intervention was not a strong predictor of the levels of inflammation and stress biomarkers at the end of the follow-up period (Table 3). Only general psychological flexibility was a significant predictor (*p* = .041) such that a higher AAQ-II score, i.e., lower psychological flexibility, predicted a higher DHEAS level. In addition, lower age (*p* < .001) and male sex (*p* = .002) were also predictors of a higher DHEAS concentration in the model.

**Table 3:** General psychological flexibility (AAQ-II, model 1) and weight-related psychological flexibility (AAQW, model 2) after the intervention as predictors of inflammation and stress biomarkers at the follow-up

	hsCRP (week 36)^a^					IL-1Ra (week 36)^a^					HMW adiponectin (week 36)^a^					Cortisol (week 36)					DHEAS (week 36)					Cortisol/DHEAS ratio (week 36)^a^				
	*b*	SE	*β*	*p*	*R*^2^	*b*	SE	*β*	*p*	*R*^2^	*b*	SE	*β*	*p*	*R*^2^	*b*	SE	*β*	*p*	*R*^2^	*b*	SE	*β*	*p*	*R*^2^	*b*	SE	*β*	*p*	*R*^2^
Model 1																														
Constant	0.55	0.46		.241	.05	2.57	0.17		*< .001*	.09	0.42	0.23		.070	.25***	70.46	37.11		.060	.02	1748.98	389.27		*< .001*	.27***	− 1.54	0.25		*< .001*	.15***
AAQ-II (week 10)^a^	− 0.07	0.23	− 0.03	.772		0.01	0.08	0.01	.888		0.01	0.11	0.01	.946		25.93	18.23	0.13	.158		395.60	191.28	0.17	*.041*		− 0.05	0.12	− 0.04	.684	
Age	− 0.01	0.01	− 0.09	.326		− 0.00	0.00	− 0.04	.668		0.01	0.00	0.15	.077		0.31	0.56	0.05	.584		− 25.88	5.89	− 0.36	*< .001*		0.02	0.00	0.34	*< .001*	
Male	− 0.32	0.14	− 0.21	*.025*		− 0.05	0.05	− 0.08	.384		− 0.38	0.07	− 0.46	*< .001*		5.08	11.15	0.04	.650		367.53	117.01	0.26	*.002*		− 0.12	0.08	− 0.14	.114	
Model 2																														
Constant	0.03	0.45		.946	.07*	2.58	0.17		*< .001*	.01	0.37	0.22		.102	.27***	60.68	35.93		.094	.04	2189.77	388.30		*< .001*	.24***	− 1.81	0.25		*< .001*	.16***
AAQW (week 10)	0.00	0.00	0.16	.099		0.00	0.00	0.02	.807		0.00	0.00	0.02	.812		0.38	0.19	0.19	.056		0.10	2.10	0.00	.963		0.00	0.00	0.13	.143	
Age	− 0.01	0.01	− 0.06	.513		− 0.00	0.00	− 0.05	.604		0.01	0.00	0.17	.052		0.53	0.56	0.09	.352		− 25.42	6.07	− 0.36	*< .001*		0.02	0.00	0.37	*< .001*	
Male	− 0.28	0.13	− 0.19	*.043*		− 0.04	0.05	− 0.07	.487		− 0.38	0.07	− 0.47	*< .001*		10.12	10.82	0.09	.352		377.86	116.94	0.27	*.002*		− 0.10	0.07	− 0.12	.177	

Linear regression models among the participants in the ACT intervention groups (*n* = 116 for AAQ-II and *n* = 117 for AAQW) are presented. *b* = unstandardized *B*, *β* = standardized beta. The significant *p* values are styled in italics

*hsCRP* high-sensitivity C-reactive protein, *IL-1Ra* interleukin-1 receptor antagonist, *HMW* high molecular weight, *DHEAS* dehydroepiandrosterone sulfate, *AAQ-II* Acceptance and Action Questionnaire (higher score indicates less general psychological flexibility), *AAQW* Acceptance and Action Questionnaire for Weight-Related Difficulties (higher score indicates less weight-related psychological flexibility)

*p < .05; ***p < .001

^a^Logarithmically transformed

### Explorative Analysis of Factors Associated with Inflammation and Stress Biomarkers

To explore the associations between psychological well-being with inflammation and stress biomarkers, considering also clinical and lifestyle factors related to low-grade systemic inflammation, we conducted post hoc, exploratory PCAs (a) of the variables measured at baseline and (b) of the changes during the entire study period.

#### At Baseline

Six principal components (PCs) emerged which explained a large proportion of the variation (61%) in the baseline data (Table 4). PC1 represented poor mental well-being; PC2 metabolic syndrome; PC3 age and diet; PC4 metabolic syndrome, weight-related psychological inflexibility, and dieting; PC5 diet quality; and PC6 physical activity (Table 4). With respect to the inflammation and stress biomarkers, higher IL-1Ra and lower HMW adiponectin levels were present with the higher BMI and higher waist circumference in PC2. A lower DHEAS level was associated with higher age, higher diet quality index, and higher fiber intake in PC3. In addition, higher hsCRP was present with lower weight-related psychological flexibility, higher BMI, higher body fat percent, and lower energy intake in PC4.

**Table 4:** Principal component analyses (PCAs) of psychological well-being and inflammation and stress biomarkers, considering also clinical and lifestyle factors related to low-grade inflammation at baseline (*n* = 204) and of the changes during the entire study period (*n* = 173)

	PC1	PC2	PC3	PC4	PC5	PC6
Baseline						
Age	0.005	− 0.046	*0.746*	− 0.081	− 0.053	− 0.172
GHQ	*0.620*	0.031	0.096	− 0.256	0.125	0.172
PSS	*0.777*	− 0.017	− 0.081	− 0.026	0.064	− 0.164
BDI-II	*0.799*	− 0.009	0.042	− 0.054	0.211	0.042
AAQ-II	*0.789*	− 0.027	− 0.038	0.056	− 0.053	− 0.226
AAQW	*0.515*	0.109	− 0.164	*0.339*	− 0.049	− 0.293
BMI	0.038	*0.773*	0.038	*0.311*	0.153	0.079
Waist circumference	0.070	*0.907*	0.085	− 0.064	0.048	− 0.205
Body fat %	0.034	0.246	0.296	*0.849*	0.224	0.133
hsCRP	− 0.175	0.263	− 0.107	*0.358*	0.062	− 0.01
IL-1Ra	− 0.047	*0.332*	− 0.14	0.122	0.001	0.157
HMW adiponectin	− 0.022	*− 0.309*	0.285	0.206	0.068	0.014
Cortisol	0.023	− 0.005	− 0.063	− 0.169	0.024	0.108
DHEAS	0.023	0.032	*− 0.566*	− 0.207	0.059	0.014
IDQ	− 0.056	0.068	*0.408*	− 0.080	*− 0.333*	0.288
AUDIT-C	0.137	− 0.021	− 0.008	− 0.086	0.080	− 0.231
Energy intake (kJ)	− 0.001	0.006	0.021	*− 0.399*	0.131	− 0.025
SAFA intake (E%)	0.169	0.075	− 0.035	− 0.092	*0.618*	− 0.110
Fiber intake (g/MJ)	− 0.025	− 0.094	*0.414*	0.026	*− 0.526*	0.094
MET	− 0.062	− 0.027	− 0.137	− 0.188	− 0.249	*0.378*
Change (from week 36 to week 0)						
Age (baseline)	0.131	0.150	− 0.099	*0.486*	− 0.107	
GHQ Δ36	*0.694*	0.009	− 0.020	0.089	*0.268*	
PSS Δ36	*0.789*	0.062	− 0.057	0.034	− 0.011	
BDI-II Δ36	*0.680*	0.024	0.129	0.121	0.067	
AAQ-II Δ36	*0.686*	0.037	− 0.026	− 0.028	− 0.128	
AAQW Δ36	*0.520*	*0.307*	− 0.035	*− 0.253*	0.051	
BMI Δ36	0.129	*0.976*	0.110	0.000	0.007	
Waist circumference Δ36	0.182	*0.739*	0.065	0.129	0.140	
Body fat % Δ36	0.030	*0.644*	0.057	0.224	0.129	
hsCRP Δ36	− 0.063	0.196	*0.274*	0.146	0.119	
IL-1Ra Δ36	0.106	− 0.016	− 0.040	0.004	0.201	
HMW adiponectin Δ36	0.022	*− 0.280*	− 0.015	0.056	0.080	
Cortisol Δ36	− 0.084	0.027	0.094	0.239	0.059	
DHEAS Δ36	− 0.008	− 0.086	0.016	*0.413*	− 0.006	
IDQ Δ36	− 0.113	0.007	− 0.169	0.086	0.214	
AUDIT-C Δ36	0.077	0.117	− 0.054	0.189	− 0.041	
Energy intake Δ36	− 0.059	0.045	*0.450*	− 0.150	− 0.025	
SAFA intake Δ36	0.047	0.060	*0.804*	0.207	0.078	
Fiber intake Δ36	− 0.046	− 0.003	*− 0.473*	0.022	0.203	
MET Δ36	− 0.018	− 0.091	− 0.065	0.162	*− 0.657*	

The components were rotated using orthogonal (varimax) rotation. The component loadings above the threshold for interpretation are styled in italics

*PC* principal component, *GHQ* 12-item General Health Questionnaire, *PSS* 14-item Perceived Stress Scale, *BDI-II* Beck Depression Inventory-II, *AAQ-II* Acceptance and Action Questionnaire (higher score indicates less general psychological flexibility), *AAQW* Acceptance and Action Questionnaire for Weight-Related Difficulties (higher score indicates less weight-related psychological flexibility), *BMI* body mass index, *hsCRP* high-sensitivity C-reactive protein, *IL-1Ra* interleukin-1 receptor antagonist, *HMW* high molecular weight, *DHEAS* dehydroepiandrosterone sulfate, *IDQ* Index of Diet Quality, *AUDIT-C* Alcohol Use Disorders Identification Test Consumption, *SAFA* saturated fat, *E%* percentage of energy, *MET* leisure time metabolic equivalent index

#### Changes During the Entire Study Period

Five PCs emerged, explaining half of the variation (51%) of the data with respect to the changes occurring during the entire study period (Table 4). PC1 represented a change in poor mental well-being, PC2 a change in weight-related psychological inflexibility and metabolic syndrome, PC3 a change in inflammation and diet, PC4 age and a change in weight-related psychological flexibility and DHEAS, and PC5 a change in psychological distress and physical inactivity (Table 4). With respect to the inflammation and stress biomarkers, a decrease in HMW adiponectin level was associated with decreased weight-related psychological flexibility, increased BMI, increased waist circumference, and increased body fat percent in PC2. Increased hsCRP levels were present with increased energy intake, increased saturated fat intake, and decreased fiber intake in PC3. In addition, an elevated DHEAS concentration was present with older age at baseline and an increase in weight-related psychological flexibility in PC4.

## Discussion

This study investigated the effects of face-to-face and mobile app ACT interventions on circulating levels of inflammatory compounds and stress biomarkers among adults with psychological distress and overweight and obesity. The face-to-face ACT intervention achieved a minor improvement in one of the studied inflammation markers, hsCRP. Psychological flexibility (general or weight-related) after the intensive intervention period was not a marked predictor of the inflammation and stress biomarker levels 6 months after the follow-up.

The ACT-based intervention included mindfulness/meditation practices, but also other ACT processes. Thus, the aim of the intervention was to increase psychological flexibility and overall well-being instead of focusing on stress reduction/management which has often been the aim in previous mindfulness interventions [44, 71]. There is some evidence that mindfulness and meditation interventions seem to decrease circulating levels of some inflammation markers and cortisol measures [44, 71]. However, because this study seems to be the first ACT-based randomized controlled intervention study investigating the effects on hsCRP, IL-1Ra, HMW adiponectin, morning cortisol, DHEAS, and cortisol/DHEAS ratio in a real-world non-clinical adult sample, the results will be discussed in relation to previous mindfulness-based interventions.

The ACT intervention delivered in the face-to-face group sessions decreased hsCRP, although the effect was seen after the follow-up period and only when the values were compared to those in the mobile ACT group and not compared to the control group. This is partly in line with previous findings from the same intervention study showing that the effects of ACT are more pronounced in the face-to-face group [48]. Although the intervention content was the same, participants in the face-to-face group were, on average, more extensively exposed to the treatment, and it is also possible that participants in the face-to-face and mobile groups have applied ACT in their personal lives differently. Furthermore, the face-to-face and mobile groups did not differ after adjusting for age, sex, and baseline BMI. This result indicates that BMI has stronger effect on the modulation of the hsCRP concentration than the intervention effect.

The within-group decrease of the hsCRP level in the face-to-face group was significant. Because hsCRP increases the risk of cardiovascular disease linearly [72], any decrease in hsCRP is beneficial. However, when adjusted for study design, age, sex, and baseline BMI, the 0.2 mg/L decrease of mean hsCRP in the face-to-face group may not be clinically significant. It is noteworthy that over a third (36%) of the participants had a low hsCRP level (< 1 mg/L) already at baseline, making it difficult to achieve a large intervention effect.

The intervention effect on hsCRP is somewhat similar to the previous mindfulness-based RCTs. No significant effect compared to control has been observed among adults with obesity [73, 74], and in healthy older adults, the hsCRP level has decreased only marginally as compared to control [75]. Although baseline BMI did not interact with the effectiveness of the ACT intervention in the present study, a mindfulness-based intervention was previously found to be effective among participants with BMI < 30 kg/m^2^, whereas there was no effect among participants with higher BMI values [73].

To the best of our knowledge, there are no previous ACT or mindfulness intervention studies reporting effects on IL-1Ra concentrations. In the present study, the levels of IL-1Ra decreased from baseline to follow-up in all study groups. A decline in the IL-1Ra concentration is considered to reflect a reduced inflammatory state of the body [76], and weight loss and dietary changes have been reported to decrease IL-1Ra levels [76]. Thus, it is surprising that in our exploratory PCA, none of the concurrent changes (i.e., changes in psychological well-being, body size and composition, other inflammation and stress biomarkers, diet quality, or physical activity) were associated with any change in the IL-1Ra level. However, there may be some underlying dietary changes reflected in the overall decrease in IL-1Ra without there being a concurrent decrease in other inflammation markers. Thus, IL-1Ra appears to be a more sensitive marker for dietary changes (e.g., in the intake of dietary fatty acids, magnesium) than, for example, the levels of hsCRP and HMW adiponectin [77, 78], also in the absence of concurrent changes in body weight [77, 78].

The observation of no effects of ACT on HMW adiponectin level is in line with previous mindfulness-based pilot studies among people with obesity (*n* = 10) [39] and among bariatric post-surgery patients being provided with a mindfulness intervention (*n* = 9) as compared to patients receiving a standard intervention (*n* = 9) [79]. Because the findings of these previous studies were only preliminary, the evidence for the effects of ACT or mindfulness-based interventions on adiponectin is still limited.

The ACT-based intervention in the present study also did not affect the stress biomarkers, i.e., cortisol and DHEAS. Previous controlled studies of mindfulness-based interventions in non-clinical samples have not influenced serum morning cortisol [80], single time point cortisol level (from 11 a.m. to 8 p.m.) [81], and the cortisol level 20–30 min after awakening [73, 82]. One meta-analysis of seven mindfulness- and meditation-based interventions reported a decrease in cortisol levels with a medium effect size [44], although another found a reducing effect for diurnal cortisol slopes but, consistently with our results, no effects on single time point measurements [71].

To the best of our knowledge, this is the first study to have investigated the effects of mindfulness- or ACT-based intervention on DHEAS and cortisol/DHEAS ratio in a non-clinical sample. The present results are in line with the study conducted in early-stage breast and prostate cancer patients, in which a mindfulness-based stress reduction (MBSR) intervention did not change the participants’ DHEAS levels or cortisol/DHEAS ratio [83].

The ACT intervention of the present study was delivered in two different ways: via face-to-face group sessions with a workbook and homework and, independently, via a mobile app. The level of psychological flexibility (general or weight-related) immediately after the intervention was used as a commensurable marker of the individual response to the ACT intervention. Psychological flexibility after the intensive intervention period did not predict the levels of inflammation markers and cortisol after the follow-up among the participants in the ACT intervention groups. However, higher general psychological flexibility predicted lower DHEAS levels, although higher age and female sex were stronger predictors in the model. DHEAS is indeed age and sex specific: the DHEAS concentrations decline during adulthood, and the levels are lower in females than in males [84].

The exploratory PCAs revealed some associations between weight-related psychological flexibility and inflammation and stress biomarkers in some of the principal components. At baseline, lower weight-related psychological flexibility was present with higher hsCRP, higher BMI, higher body fat percent, and lower energy intake reflecting the presence of metabolic syndrome and either dieting or dietary under-reporting. This result is logical because lower weight-related psychological flexibility, i.e., avoidance and inflexibility related to difficult weight-related thoughts and feelings, has been associated with higher BMI values [54] which, in turn, elevate the CRP level [22].

Furthermore, changes in weight-related psychological flexibility were associated with changes in some of the inflammation and stress biomarkers. Although an increase in weight-related psychological flexibility was not associated with a change in the hsCRP level, it was associated with other features of the metabolic syndrome (i.e., a decline in BMI, a reduction in waist circumference, a decrease in body fat percent, and an increase in HMW adiponectin level) in one principal component. Thus, the increase in weight-related psychological flexibility was associated with positive changes in inflammatory status and body composition. Among older participants, the increase in weight-related psychological flexibility was also associated with a positive change, namely an increase in the DHEAS level.

Surprisingly, cortisol levels were not associated with any subjective measures of distress or with the DHEAS levels at baseline or, longitudinally, in our exploratory PCAs. While the cortisol level is elevated in an acute stress response [14], it can be either increased or decreased in chronic stress situations [11]. Cortisol secretion seems to depend on the time elapsing since stress exposure onset, the nature of the stressor, and personal factors [11], in addition to the amount of visceral fat [13]. Thus, for some study participants, the chronic stress response may have been reflected in an increased morning cortisol level while for others, it may have resulted in a decreased cortisol level, making it impossible to detect clear associations with other studied variables. Furthermore, cortisol secretion follows a distinct diurnal rhythm [85] which also may have affected the present results because although all the blood samples were drawn in the morning, it was not possible to have study participants visiting the laboratory at precisely the same time after awakening on each study visit. Thus, utilizing a single time point for blood samples for cortisol measurement is a limitation in the current study [86]. Furthermore, the use of the AAQ-II may have limited our ability to assess general psychological flexibility, because the validity of AAQ-II is questionable [87, 88].

Another limitation is that, as this was a secondary analysis of the intervention study, the analyses for intervention effects may have been underpowered. Furthermore, we did not take into account the possible influence of menopausal status or medication as covariates [89]. It is also noteworthy that the PCA used in our explorative analyses has limitations. PCA was used to explore how inflammation and stress biomarkers would associate with psychological, anthropometric, and lifestyle measures since all of these are claimed to be intertwined. Our intention with PCA was to identify which variables would emerge with inflammation and stress biomarkers in the principal components. However, most of the variance of the data was explained by the psychological variables. Furthermore, some of the loadings of the rotated components were rather low, and thus, the PCA results regarding inflammation and stress biomarkers should be interpreted with caution. The dietary intake measured with a retrospective 48-h dietary recall is also a limitation. The outcome depends on participants’ memory. However, this was addressed in our interview protocol [62] that was based on the protocol used in national dietary intake study [61]. The 48-h dietary recall was used instead of, e.g., food records to diminish the burden on the participants and because also the other methods have limitations [90].

There are also several strengths in the present study. The intervention study was conducted as a randomized controlled trial with a rather large sample size. Working-age participants with subjective psychological distress and overweight were recruited, meaning that our participants had a high risk for low-grade systemic inflammation and metabolic syndrome. Biomarkers were measured in the fasting state, and the main statistical analyses were controlled for age, sex, and BMI. To the best of our knowledge, this is the first study to investigate the effects of ACT on circulating inflammation and stress biomarkers in a non-clinical adult sample in a real-world setting. Furthermore, the associations between psychological flexibility and these biomarkers have not been studied before. Thus, the present results provide insights into how these two underlying process measures associate with each other: the psychological process behind human suffering and the physiological process behind ill health.

In psychological science, there is a move towards process-based care to target the processes underlying human suffering irrespective of diagnoses (i.e., transdiagnostic perspective) [31, 36, 91]. Transdiagnostic, process-based care makes it possible to affect several diseases and symptoms without focusing directly on those diseases or symptoms. This may be extremely helpful among people with overweight and psychological distress, because obesity is closely linked to weight stigmatization (i.e., prejudice, discrimination, and negative attitudes towards people with obesity), which, in turn, contributes to stress and obesity [92]. Utilizing process-based psychological care may represent one way to help these individuals, e.g., to reduce low-grade systemic inflammation and the risk of type 2 diabetes without focusing on obesity. However, these primary results highlight that more research is needed on how best to apply the process-based care for the health of both mind and body among people with overweight and psychological distress.

In conclusion, these preliminary results suggest that a general ACT intervention delivered in face-to-face group sessions may have some beneficial effects on inflammation. Weight-related psychological flexibility may be a feature of the psychological processes linked to certain physiological processes such as low-grade systemic inflammation. Further studies of ACT interventions are needed to target specific physiological pathways by means of psychological processes (i.e., psychological flexibility).
